# Non-Tuberculous Mycobacterium: A Rare Cause of Granulomatous Hepatitis

**DOI:** 10.4021/gr538w

**Published:** 2013-05-03

**Authors:** Chijioke Enweluzo, Anuradha Sharma, Stephen Lenfest, Fahad Aziz

**Affiliations:** a Section on Hospital Medicine, Department of Internal Medicine, Wake Forest Baptist University Medical Center, Medical Center Boulevard, Winston Salem, NC 27101, USA; b Section on Infectious Diseases, Department of Internal Medicine, Wake Forest Baptist University Medical Center, Medical Center Boulevard, Winston Salem, NC 27101, USA; c Department of Pathology, Wake Forest Baptist University Medical Center, Medical Center Boulevard, Winston Salem, NC 27101, USA

**Keywords:** Granulomatous, Hepatitis, Nontuberculous, Mycobacterium, Clarithromycin, Fibrin-ring, Fever of Unknown Origin, Chelonae-Abscessus

## Abstract

Granulomatous hepatitis is a syndrome usually characterized by fever of unknown origin, myalgias, hepatosplenomegaly, and arthralgias, right upper quadrant abdominal pain or tenderness, with or without an elevation in serum transaminases. In this article, we outline our experience with a 64-year-old male presenting with a 3.5 weeks history of fever of unknown origin, night sweats, extreme fatigue and a 20 lb. weight loss. He had an extensive evaluation including 2 liver biopsies that was indicative of fibrin ring granulomas and a positive PCR for Mycobacterium chelonae-abscessus. He was eventually treated empirically with antibiotics that led to an improvement of his symptoms.

## Introduction

Granulomatous hepatitis is a syndrome usually characterized by fever of unknown origin (FUO), myalgias, hepatosplenomegaly, and arthralgias, right upper quadrant abdominal pain or tenderness, with or without an elevation in serum transaminases [1]. FUO represents a diagnostic challenge for any clinician. This is probably further worsened by an additional constellation of signs and symptoms as described in our case below. Many causes of granulomatous hepatitis have been identified. Our experience brings to light a rare infectious cause which if considered early on in the disease course could have prevented unnecessary invasive measures and ensured better health results.

## Case Report

We describe the case of a 64-year-old Caucasian male who was transferred to our facility for evaluation of a 3.5 weeks history of fever of unknown origin, night sweats, extreme fatigue and a 20 lb. weight loss. He saw his PCP at the onset of his symptoms and was given Doxycycline. However, he continued to experience a daily fever spike in the mornings and evenings in addition to worsening fatigue. This prompted presentation to an outside hospital. Initial labs at the outside hospital showed transaminitis with AST/ALT 109/128 and Alkaline Phosphatase 182. A laparoscopic cholecystectomy and appendectomy for suspected symptomatic cholelithiasis/appendicitis was performed. A liver biopsy was also obtained during the procedure. Following surgery, he was afebrile for a day but spiked a fever of 40.6 °C while receiving empirical antimicrobial coverage with Vancomycin, Doxycycline, Zosyn and Azithromycin over 2 days without much improvement in his symptoms. Subsequently, he was transferred to our facility for further evaluation and management.

**Figure 1:**
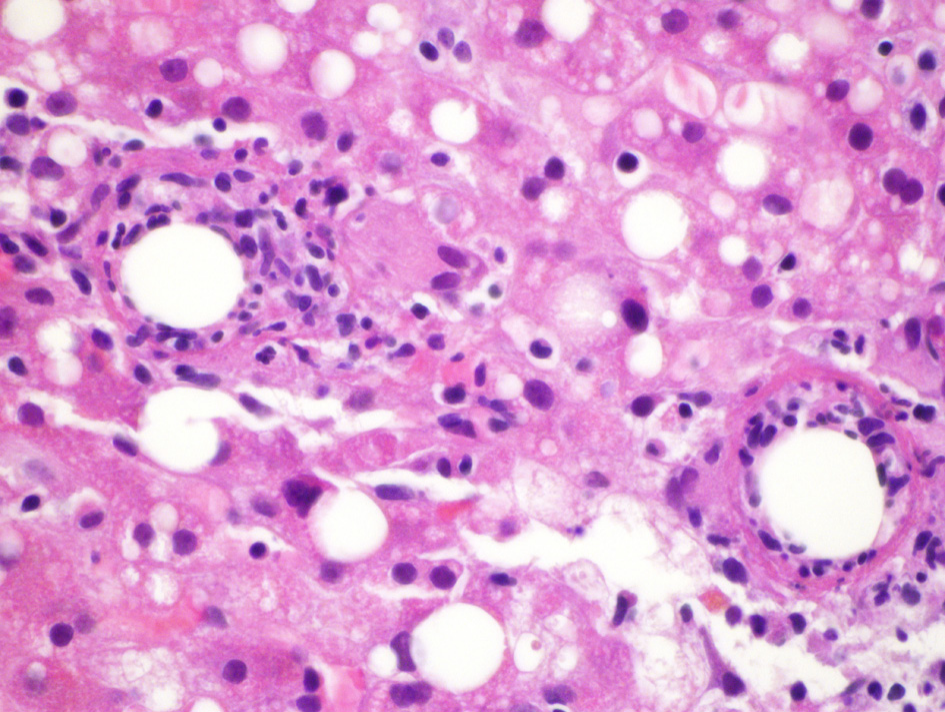
Initial liver biopsy showing a background of steatohepatitis with 2 adjacent fibrin ring granulomas (H&E × 600).

**Figure 2:**
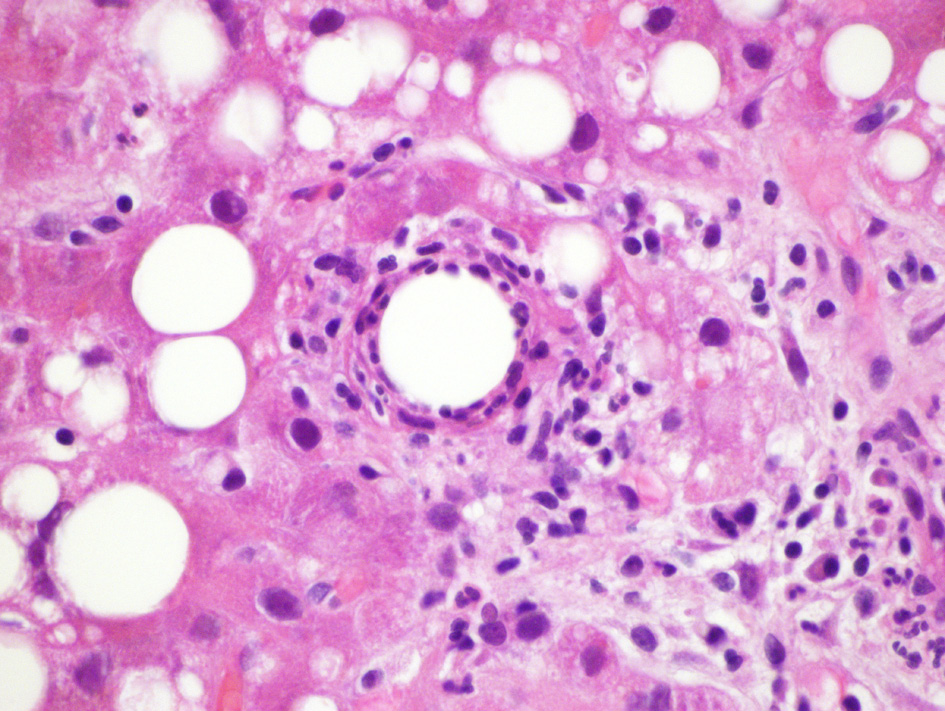
Fibrin ring granuloma from second liver biopsy (H&E × 600).

On admission to our facility, he still had the same complaints and was found to be febrile, icteric and uncomfortable on physical examination. Lab work was significant for AST 75, ALT 97 and total bilirubin 5.3. An abdominal ultrasound showed hepatosplenomegaly, hepatic steatosis, and post-surgical changes from his recent cholecystectomy without fluid collection. 2D ECHO was normal. Serology workup included: CMV IgM equivocal, CMV IgG positive, but CMV PCR of 2685 copies, EBV-PCR was negative, C2, C3, C4 were normal, CH50 was 15(low), ferritin 776, ESR 32, CRP 184.6. ANA screen was positive with titer 1280, ANCA and Anti Smith IgG were negative. HIV ab was negative. Leptospira and Coxiella antibodies were negative. Brucella PCR was negative, serology was equivocal at 1:20 but the patient did not have any epidemiologic risk factors. RMSF titer and PPD were also negative. The specimen slides of the liver biopsy performed during the cholecystectomy at the initial hospital were submitted to the CDC for review (Fig. 1). A second liver biopsy was performed at our facility (Fig. 2). Pathology from both procedures revealed a background of steatohepatitis with numerous fibrin-ring granulomas scattered throughout characteristic of granulomatous hepatitis (Fig. 1, 2). The CDC also reported PCR positive for Mycobacterium chelonae-abscessus. PCR for brucella and coxiella were negative. Given that the only positive work up was a positive PCR for Mycobacteria abscessus-chelonae, we elected to empirically treat him with clarithromycin, moxifloxacin and minocycline on discharge. Two weeks later, he was seen in clinic where he denied any fever or chills since discharge but complained of continued fatigue, weight loss and general deconditioning. We continued him on the same antibiotics. A few weeks later, he reported weight gain of about 7 pounds and was no longer fatigued. He was able to return to his usual activities like gardening and housework. He also denied any fevers, night sweats or chills. Considering his positive ANA antibody screen, he was evaluated by gastroenterology as well as ophthalmology for autoimmune diseases. There was no clinical, serological or radio graphical evidence of Sarcoidosis or autoimmune hepatitis.

## Discussion

The term “Granulomatous hepatitis” is often used, but the condition has not been defined as a true hepatitis. Considering the nonspecific nature of this condition in terms of presentation and lab findings as well as broad etiologies, diagnosis can be challenging and altogether missed in many cases. A liver biopsy is essential and could be helpful in diagnosis. However, in many cases, biopsies can be normal. Hepatic granulomas are found in about 3 to 10% of liver biopsies [2]. Many causes of Granulomatous Hepatitis have been identified and grouped into 5 broad categories including [3]: 1). Systemic Infections such as HIV/AIDS, CMV, Q fever, and Brucellosis, fungal diseases, Tuberculosis and infections from nontuberculous mycobacterium (NTM); 2). Drugs such as Allopurinol, Sulfa, Chlorpropamide, Quinidine, etc; 3). Malignancy such as Hodgkin’s and Non-Hodgkin’s Lymphoma, Renal cell carcinoma, etc; 4). Autoimmune disorders such as Sarcoidosis and Primary Biliary Cirrhosis; 5). Idiopathic.

Across all categories, cases due to Sarcoidosis and Tuberculosis have the highest incidences. However, lesser-known causes such as mycobacterium are rarely encountered and have a sparse mention in published literature. Mycobacterium chelonae-abscessus is a nontuberculous mycobacterium grouping that encompasses all mycobacteria outside of the Mycobacterium tuberculosis complex [4, 5]. They are classified in Runyon group IV, rapidly growing mycobacteria. M chelonae is found in natural and processed water sources, including sewage, and especially in tap water [4, 5]. Mycobacterium abscessus and M chelonae share similar genetic backgrounds and many reports have simply characterized the two species together. For this reason, determining which species is actually involved in an infection is often difficult [5]. Members of the M. chelonae-abscessus complex represent Mycobacterium species that cause invasive skin and soft tissue infections, pneumonia, bloodstream infections, and abscesses in immunocompetent and immunocompromised hosts [5]. Patients infected with M. chelonae-abscessus present with fever, easy and prolonged fatigability, night sweats, and weight loss occurring with pulmonary or disseminated disease [6]. Prolonged antibiotic therapy is generally considered for M chelonae infections. Many reports have documented cases of successful therapy with Clarithromycin; however, other reports have described the development of resistance to monotherapy. Antibiotic therapy with 2 drugs is preferable in most patients. No standard duration of therapy is reported. Treatment usually lasts for many months, and courses that are 6 months or longer are not unusual [7, 8].

Our patient presented with fever of unknown origin among other symptoms. Two separate liver biopsies, one of which was reviewed at the CDC, showed granulomatous hepatitis with fibrin ring granulomas. Extensive work up including tissue cultures and AFB as noted above were negative except for a positive PCR for Mycobacterium chelonae spp abscessus on the liver biopsy. However, it is pertinent to note that doxycycline administered early on in our patient’s course by his PCP may have suppressed M. chelonae-abscessus growth on culture. He did not show any evidence of disseminated atypical mycobacterial disease but following our decision to empirically treat him for the same, he demonstrated marked clinical improvement and stabilization of his transaminases.

### Conclusion

Our case highlights the importance of recognizing the entity of non-tuberculous mycobacteria infection as a cause of granulomatous hepatitis especially in patients presenting with fever of unknown origin and signs of liver damage. This will lead to proper early treatment, resolution of liver disease as seen in our patient and prevent unnecessary invasive measures such as cholecystectomy and/or liver biopsy.
